# Curvisetone—A Male-Specific Tricyclic *nor*-Diterpenoid from the Springtail *Sinella curviseta*

**DOI:** 10.1021/acs.jnatprod.4c01432

**Published:** 2025-03-12

**Authors:** Anton Möllerke, Stefan Schulz

**Affiliations:** † Technische Universität Braunschweig, Institute of Organic Chemistry, Hagenring 30, 38106 Braunschweig, Germany

## Abstract

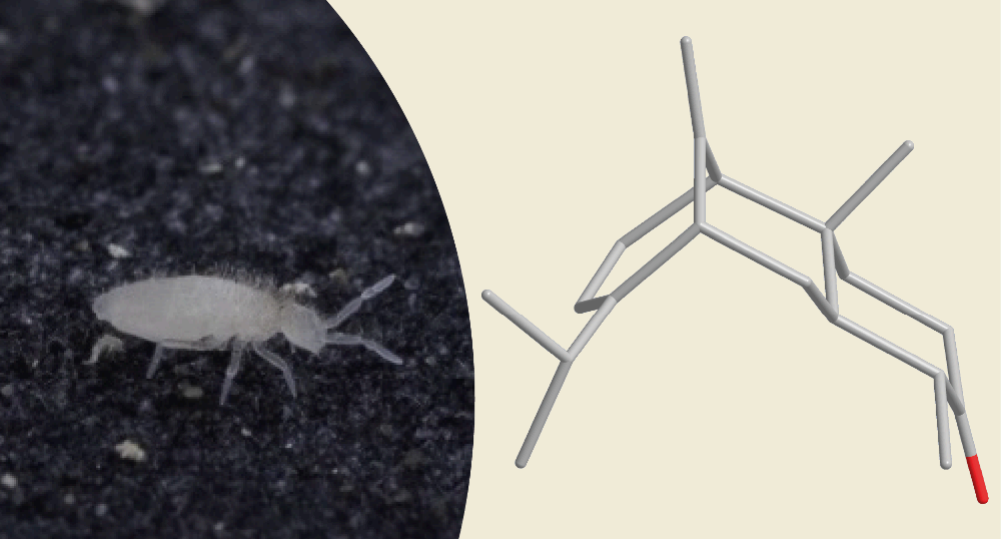

The structure determination and occurrence of curvisetone (**1**), a tricyclic nor-diterpenoid from the tiny Collembola *Sinella curviseta*, is reported. Utilizing NMR analysis and mass spectrometry, we identified curvisetone’s unique carbon skeleton and its occurrence in various sex and life stages. Curvisetone, carrying an unprecedented carbon tricyclic ring system, is an example of the distinct terpene usage of Collembola, which differentiates them from other arthropods.

Springtails (Collembola) represent the most abundant soil arthropods in many soil ecosystems, especially in temperate regions.^1^ Despite their importance for soil health, a sufficient understanding of their secondary metabolites and chemical ecology, e.g., their intraspecific and interspecific chemical mediated interactions, is still lacking. This may be due to their tiny size and often long lifecycles, which makes research on their natural products tedious. However, recent research centered on the composition of the epicuticular wax, chemical defense, and, in a few cases, signaling compounds.^2^

Especially the wax components are often terpenes with unique structures, such as linear, fully head-to-tail connected hepta-, octa-, and nonaprenyls with cyclized head groups such as poduran and decahydropentaprenylprespatane from *Podura aquatica*.^3,4^ Open-chain compounds with prenyl or geranyl side chains, as in viaticene or nitidane, also occur.^5^ As volatile terpenoids, so far only geosmin,^6^ a soil bacterial compound known to attract springtails,^7^ and the diterpene sclareol were reported from *Ceratophysella sigillata* and *Folsomia quadrioculata*, respectively.^4^
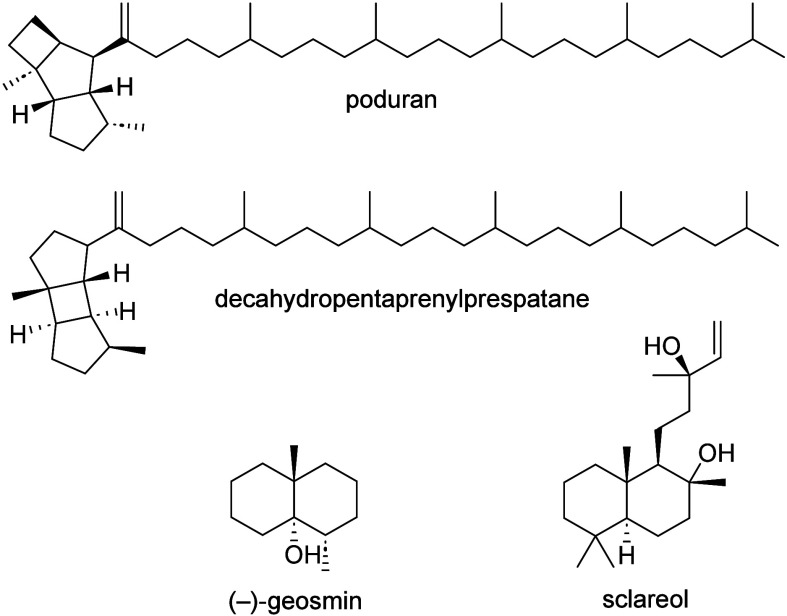


*Sinella curviseta* occurs widespread in the Northern Hemisphere and serves as a model organism in soil ecology and ecotoxicology. Females of *Sinella curviseta* produce a volatile pheromone which increases spermatophore deposition of conspecific males.^8^ These data inspired us to look into the production of volatiles potentially involved in communication in this species.

While analyzing a whole-body pentane extract, we detected major compound **1** (gas chromatographic retention index *I* 2023, HP5-MS phase) with a mass spectrum not similar to any compound in our extensive databases (Figure 1).

**Figure 1:**
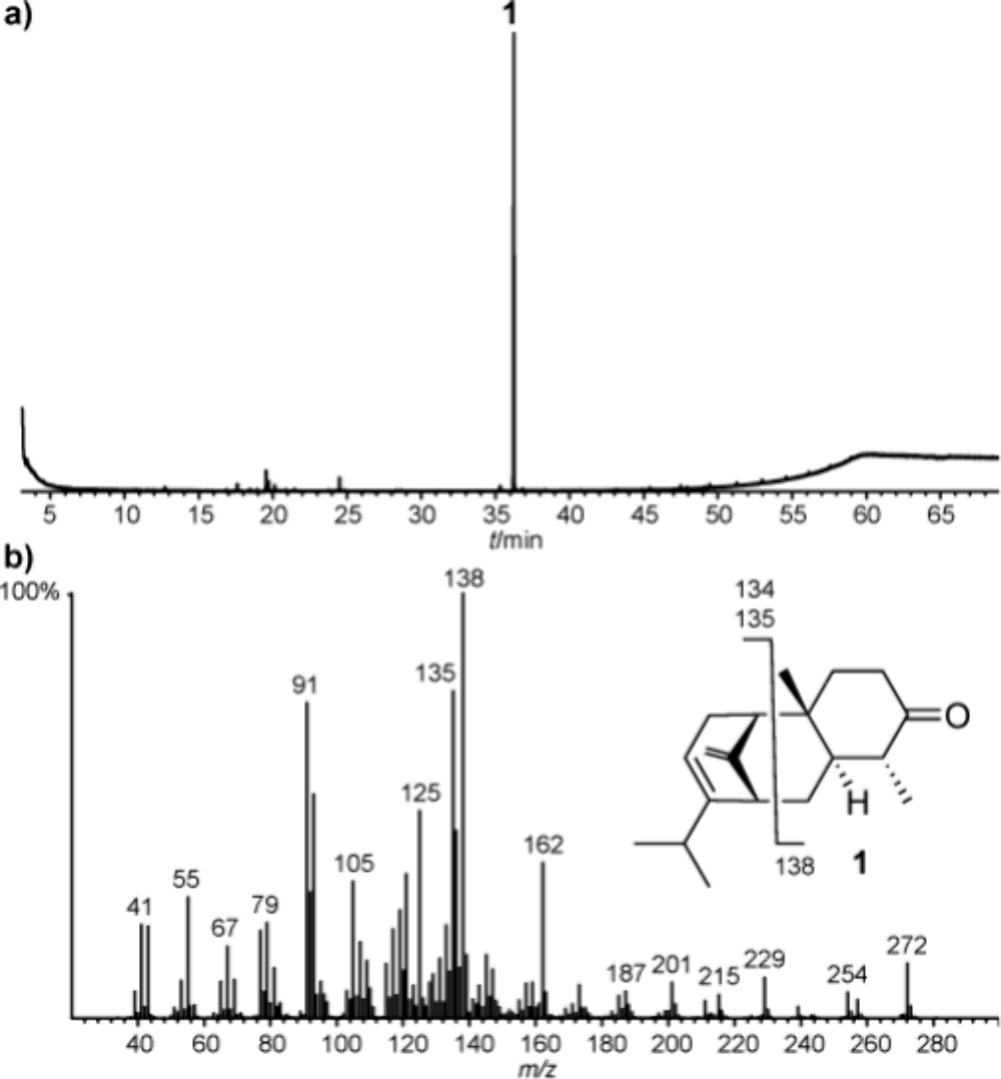
TIC of a pentane extract of *S. curviseta* (a) and mass spectrum of compound **1** (b).

High resolution-MS (HR-MS) gave a molecular ion of *m*/*z* 272.2138, which translates into the molecular formula C_19_H_28_O (calcd. for C_19_H_28_O 272.2135) with six ring double bond equivalents (rdbe). GC/IR analysis showed an intense band at 1709 cm^–1^, which aligns with a ketone. Treatment of the extract with Pd/C and H_2_ delivered a mixture of two compounds of the same molecular ion at *m*/*z* 276 (Figure S1). HR-MS showed that both compounds have a molecular formula of C_19_H_32_O (*m*/*z* 276.2448 and 276.2447; calcd. for C_19_H_32_O 276.2448), which indicates two double bonds. The remaining three residues indicate a tricyclic structure of compound **1**.

More biological material was needed to elucidate the structure of compound **1**. Therefore, we cultured *S. curviseta* for several months, yielding about 5 g of Collembola. Extraction and micro flash chromatography yielded 0.43 mg of compound **1** (0.43 mg), which was analyzed using different NMR experiments (Table 1).

**Table 1:** ^1^H-NMR (600 MHz, CDCl_3_) and ^13^C-NMR (150 MHz, CDCl_3_) as well as HMBC and NOESY Signals of Compound **1**

Position	C [ppm], type	H [ppm], m (*J* [Hz])	HMBC	NOESY
1a	38.47, CH_2_	2.57, tdd (14.2, 6.5, 1.0)	2, 12	1b, 3, 12b, 19
1b		2.40, m	2, 3, 12	1a, 3, 12a, 12b
2	213.12, C			
3	45.08, CH	2.24, m	2, 4, 5, 11, 15	1a, 4, 5b, 15, 19
4	42.09, CH	1.69, m	3, 5, 19	3, 9a, 10, 12b, 15
5a	32.04, CH_2_	1.65, m	4, 11, 13	5b, 6, 12b, 15, 17
5b		1.44, m	4, 7	5a, 6, 19
6	42.21, CH	2.82, m	4, 5, 7, 8, 10, 13, 14, 16	5a, 5b, 14a, 16, 17
7	146.29, C			
8	119.52, CH	5.38, t (3.5)	6, 10, 16	4, 9a, 9b, 17, 18
9a	28.78, CH_2_	2.39, m	7, 8, 10, 11, 13	8, 9b, 10, 12a
9b		2.16, m	7, 8, 11	8, 9a, 10
10	49.08, CH	2.02, d (6.5)	4, 6, 8, 9, 11, 13, 14, 19	9b, 14b, 19
11	38.67, C_q_			
12a	36.27, CH_2_	2.05, m	1, 11, 19	1a, 1b, 4, 9b, 12b
12b		1.41, m	1, 2, 11, 19	1b, 9b, 10, 12a, 19
13	150.65, C			
14a	104.18, CH_2_	4.75, d (2.2)	6, 10, 13	14b, 6, 19
14b		4.60, d (2.2)	6, 10, 13	14a, 10, 19
15	11.50, CH_3_	0.92, d (6.5)	2, 3, 4	3, 4
16	33.18, CH	2.12, m	6, 7, 8, 17, 18	6, 8, 17, 18
17	22.50, CH_3_	0.99, d (6.6)	7, 16, 18	5a, 6, 8, 16, 18
18	21.57, CH_3_	1.00, d (6.6)	7, 16, 17	6, 8, 16, 17
19	18.36, CH_3_	1.12, d (0.7)	4, 10, 11, 12	1a, 3, 5b, 10, 12b, 14a, 14b

The carbonyl carbon at 213.12 ppm (C-2) aligns with a ketone. Furthermore, two alkenes can be identified: a trisubstituted alkene with signals at 146.29 ppm (C-7, C) and 119.52 ppm (C-8, CH), as well as a disubstituted, terminal alkene at 150.65 ppm (C13, C) and 104.18 ppm (C-14, CH_2_). The protons H_2_-1, H-3, H_2_-12, and H_3_-15 show HMBC correlations with the carbonyl carbon (C-2). The methyl group (H_3_-15) shows a COSY correlation to H-3 (*J* = 6.5 Hz), indicating that CH-3 binds to C-2 and CH_3_-15. The two methylene groups, H_2_-1 and H_2_-12, correlate in the COSY but not to other nuclei (*J* = 14.2 Hz). Given the higher downfield shifts of CH_2_-1 over CH_2_-12 and the HMBC correlation of H_2_-1 to C-3, we concluded CH_2_-1 to be the direct neighbor of the ketone (C-2). Besides H_3_-15, there is an additional COSY correlation of H-3 to H-4; following the chain, H-4 correlates to H_2_-5, which correlates to H-6, which does not show other correlations. The groups CH-4 and CH-6 need to bind to one and two additional non-hydrogenated carbon centers, respectively. The H-4 shows HMBC correlations with quaternary carbon C-11, indicating neighbors. The multiple HMBC correlations of H-6 include the non-hydrogenated carbons C-7 and C-13, indicating it to be a major branching point. The carbon C-7 is connected to the isopropyl group (CH-16, CH_3_-17, CH_3_-18) and CH-8. The alkene proton H-8 shows a COSY correlation with H_2_-9 (*J* = 3.5 Hz), which also correlates with H-10. The absence of further COSY correlations of H-10 to other groups indicates two neighboring non-hydrogenated carbon centers. Furthermore, H-10 shows HMBC correlations with C-11 and C-13 and a weak ^4^*J* correlation with C-7. From these data, the A-ring was established to be a 1,3-bridged cyclohexene. The HMBC correlation of H-4 and H-10 with C-11 solves the B-ring. Further HMBC correlations of H_3_-19 and H_2_-12 with C-11 completed the constitution of **1** (Figure 2).

**Figure 2:**
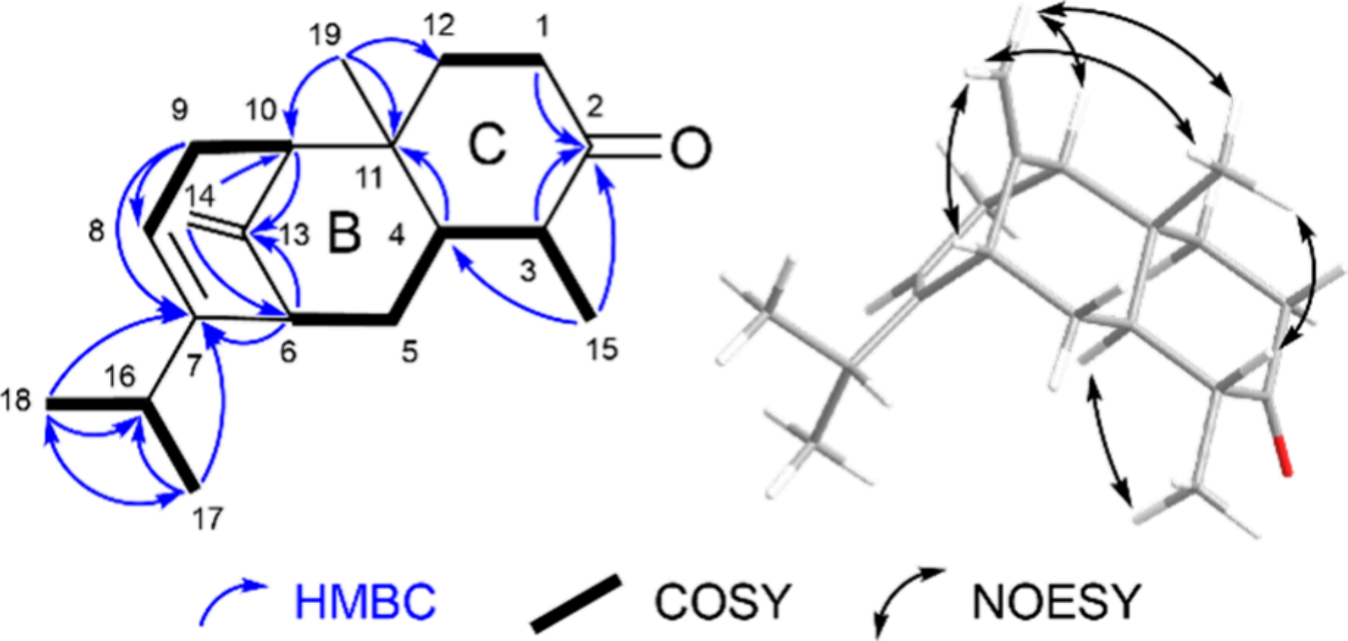
Left: Structure of compound **1** with key COSY and HMBC correlations (CDCl_3_, ^1^H: 600 MHz; ^13^C: 150 MHz). Right: Geometry optimized by the DFT (ωB97X-D; 6-31G*) structure with NOESY correlations.

The NOESY correlations reveal the relative configuration of compound **1** (Figure S7). A correlation of H-8 and H-16 shows the isopropyl group and the alkene proton to be *cis-*configured. The protons H-14a and b of the terminal alkene correlate with H_3_-19, only H-14a correlates to H-6, and only H-14b correlates to H-10. This shows that the methyl group CH_3_-19 is on the same side of the ring as the bridging group C-13. Furthermore, the protons H-6 and H-10 are facing toward the bridge. The methyl group H_3_-19 correlated to H-3 but not H-4, indicating the relative configuration shown in Figure 2.

The geometry of the proposed structure was optimized, and NMR data were calculated by density functional theory (DFT) (ωB97X-D; 6-31G*, see Table S1). The proposed geometry aligned with the experimental NOESY signals as all correlating nuclei were less than 4 Å apart. The comparison of calculated and experimental NMR data gave a root-mean-square error of 1.34 ppm (^13^C) and 0.12 ppm (^1^H). Furthermore, the IR spectrum was calculated using DFT methods (B3LYP; 6-311+G(d,p)) with the optimized geometry. The calculated IR spectrum aligns well with an experimental spectrum of **A** (Figure S3).

The GC/MS and GC/IR data are consistent with the proposed structure. The mass spectrometric fragmentation of compound **1**, which we call curvisetone, is shown in Figure 1. Measurements of the specific rotation failed due to the low concentration; hence, elucidating the absolute configuration was impossible.

With the structure of curvisetone in hand, we became interested in its functional role in the physiology of *S. curviseta* and its occurrence in different life stages and sexes. Using eggs, we started synchronized colonies of which a small number of springtails were removed for analysis every few days, and their CH_2_Cl_2_ extracts were analyzed by GC/MS (Figure S4). More animals were used in the early life stages due to lower body mass than in later life stages. In all extracts, squalene was the most abundant natural compound. We assumed its production to be relatively stable and used it as a standard to measure the relative concentrations of **1** (Figure 3). Previously, it was reported that *S. curviseta* reaches sexual maturity after 34 days at a temperature of 20 °C or after 31 days at 24–25 °C.^9^ Interestingly, curvisetone (**1**) was absent in very young juveniles and first detected in 22-day-old animals. The relative concentration peaked in 26-day-old animals. In 37-day-old animals, the concentration of **1** reached a local low; this was coincidental with the first oviposition. Four days later, a slight increase in the concentration of **1** was detected. Due to the hatching of the eggs, no further data points were collected as a life cycle was completed. Curvisetone was only detected in older animals, reaching its highest relative concentration approximately 10 days before oviposition and reaching its lowest relative concentration the day of oviposition.

**Figure 3:**
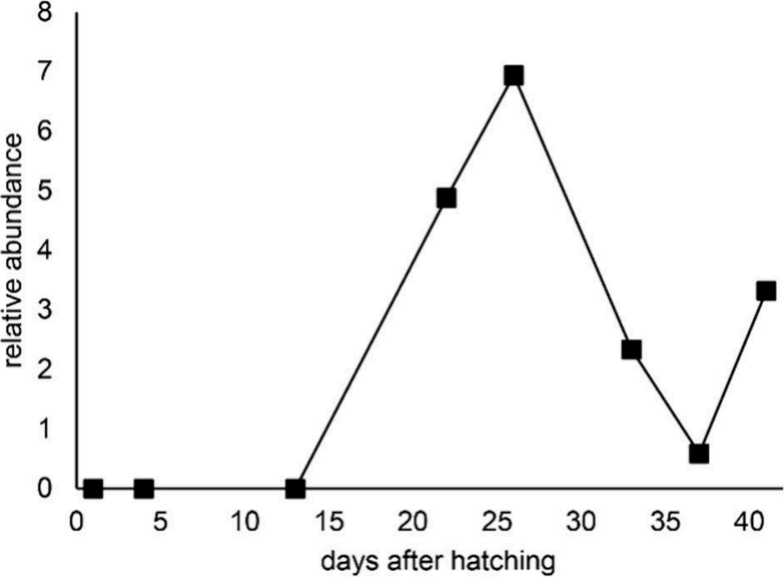
Relative abundance of curvisetone (**1**) in TICs of CH_2_Cl_2_ extracts of *S. curviseta* over time, in days after hatching. The abundance is given as the relative peak area of **1** versus that of squalene set to 100%.

We then extracted and analyzed females and males separately. Sexing was only roughly achieved using the different body mass of the sexes as an indicator.^9^ However, both sexes showed significantly different abundances of curvisetone (**1**). The relative peak ratio of squalene and **1** in females was 100:5.7 (*N* = 4), while, in males, a ratio of 41.6:100 (*N* = 4) was found. Effectively, males have roughly 40 times higher concentrations of **1** than females.

The changes in the relative concentration of curvisetone in the course of the reproductive phase, as well as the significant sex differences in abundance, may indicate the biological function of curvisetone (**1**) to be related to reproduction, e.g., attracting females, synchronizing reproductive behavior of the colony, or increasing or inhibiting spermatophore deposition. Waldorf had reported that spermatophore deposition behavior is increased by both chemical cues of conspecific females and antenna amputation, although we found a male-specific occurrence here.^8,10^ A spermatophore attractant for females may also be possible, although the only known sperm attraction pheromone, (*Z*)-14-tricosenol of *Orchesella cincta*, is biosynthesized via a fatty acid pathway.

## Conclusions

The structure of curvisetone (**1**) was elucidated to be (1R*,4aS*,5S*,9S*,10aR*,11S*)-8-isopropyl-1,4a,11-trimethyldecahydro-5,9-methanobenzo[8]annulen-2(1*H*)-one. This tricyclic carbon skeleton was, to the best of our knowledge, not reported before; therefore, it adds to several unprecedented terpenes reported from Collembola. The male-specific abundance of curvisetone and changing abundance during the life cycle of *S. curviseta* may indicate an involvement in reproductive behavior.

## Experimental Section

### General Experimental Procedures

IR spectra were measured on an Agilent Technologies 7890B gas chromatograph with an HP5 phase connected to a Dani Instruments DiscovIR DDFTIR-Interface. NMR spectra were recorded on an AVNeo600 (^1^H NMR: 600 MHz, ^13^C NMR: 150 MHz) instrument. All NMR spectra were referenced to tetramethylsilane at 0.00 ppm. Mass spectra were recorded with a combination of an Agilent Technologies 5977B gas chromatograph connected to an Agilent Technologies 8860 Series MSD. Gas chromatographic retention indices were calculated against a series of *n*-alkanes according to van den Dool and Kratz^11^ using a standard HP-5 phase. An Exactive GC orbitrap mass spectrometer (ThermoScientific, Bremen, Germany) was used for high-resolution MS. The resolution was set to 60,000 (fwhm; instrument setting at 200 u). The mass range was 50–650 u, and two micro scans were averaged per data scan. Automated gain control (AGC target) was set to 1 × 10^6^, and the maximum inject time was set to “auto”. Auxiliary temperatures were set to 290 °C for transfer lines 1 and 2, and the temperature of the electron ionization source was set to 220 °C. EI was performed at 70 eV energy in the positive mode. Helium (carrier gas) and nitrogen (supply for C-Trap) were equipped with gas purification cartridges to trap moisture and organic impurities of the gases (Thermo Scientific, Bremen, Germany). Column bleed ion at 207.03235 u was used as lock mass for internal mass calibration of the data. For chemical ionization in positive mode (CIP), methane (99.995%) was used as CI-gas at a 1.5 mL/min flow rate. Column chromatography: silica 60 (0.063–0.200 mm, 70–230 mesh ASTM).

### Biological Material and Isolation

*Sinella curviseta* was cultured in the laboratory for several months, yielding about 5 g of Collembola. A mixture of plaster of Paris and activated charcoal (10:1) was used as the substrate in Petri dishes, and from time to time, it was moistened with drops of water. Food (baker’s yeast) was given *ad libitum*. A GC/MS analysis of yeast extracts did not reveal the presence of any of the compounds discussed in the manuscript. The Collembola were extracted with CH_2_Cl_2_ (SupraSolv, Merck). Compound **1** was isolated by micro flash chromatography (pentane → pentane/CH_2_Cl_2_; 5:1, SupraSolv, Merck) and analyzed by NMR (Table 1).

When analyzed separately, males and females were differentiated by weight, as *S. curviseta* females reportedly have approximately twice the weight of conspecific males.^9^ To produce synchronized colonies, the substrate was checked daily for eggs; when eggs were found, all Collembola were transferred into a container with fresh substrate. The container with the eggs was again checked daily for hatched Collembola. All Collembola hatched over 2 days, and these were considered synchronized colonies. All parameters, such as light, food availability, and moisture, were kept constant.

### Microderivatization—Hydrogenation

A solution of the extract in pentane (100 μL) with a concentration suitable for GC analysis was placed in a 1.5 mL vial equipped with a 200 μL insert. A minute amount of Pd/C was added. A hydrogen atmosphere was applied for 1 h or until GC/MS analysis showed complete conversion. The catalyst was then removed by filtration through a Celite pad and directly used for GC/MS analysis.

### Computational Methods

All molecular mechanical calculations were performed using Spartan ’18 (Version 1.4.4). All conformational searches used the MMFF force field method^12^ and were calculated for the gas phase, with a 10 kJ mol^–1^ upper energy limit. Typically, the lowest energy conformer was 4–5 kJ mol^–1^ lower than other conformers, so the analyses could be simplified by discounting the higher energy conformers. The geometry optimization and NMR calculation were also performed using Spartan ’18 (Version 1.4.4). The ωB97X-D functional and the 6-31G* basis set were employed for this.

DFT-based calculations of IR spectra were performed as described previously.^13^ Quantum mechanical calculations were carried out using Gaussian 09^14^ and employed the B3LYP functional^15^ and 6-31G(d,p) basis set for all calculations using the optimized geometry. The calculated IR spectra were scaled by a factor of 0.97.

## Data Availability

The raw NMR data for curvisetone has been added to the Supporting Information. The mass spectrum of curvisetone will be included in the open-access MS database MACE^16^ after publication.

## Supporting Information Available

Calculated and experimental NMR spectra for curvisetone, mass spectra, chromatograms, calculated and experimental IR spectra, and additional experimental details (PDF)
Raw NMR data (ZIP)
