# Supplementary material for: Curvisetone—A Male-Specific Tricyclic nor-Diterpenoid from the Springtail Sinella curviseta
Source: J Nat Prod. 2025 Mar 12;88(3):857–61. doi: 10.1021/acs.jnatprod.4c01432 (PMC11959583; doi:10.1021/acs.jnatprod.4c01432)
Supplement: Supplementary file 1 — np4c01432_si_001.pdf [file np4c01432_si_001.pdf]

Curvisetone – A Male-Specific Tricyclic *nor*-Diterpenoid from the Springtail *Sinella curviseta*

*Anton Möllerke, Stefan Schulz**

[1]TU Braunschweig, Institute of Organic Chemistry, Hagenring 30, 38106 Braunschweig, Germany

Table of Contents:

1. High Resolution Mass Spectra of Compound **1** and Hydrogenation Products 2

2. Mass Spectra of Hydrogenated Compound **1** 3

3. Experimental and Calculated IR data 4

4. Time Course of Curvisetone Production 5

5. DFT-calculated NMR Data 6

6. NMR Spectra 7

7. Peak List of the Mass Spectrum of Curvisetone 12

1. High Resolution Mass Spectra of Compound 1 and Hydrogenation Products

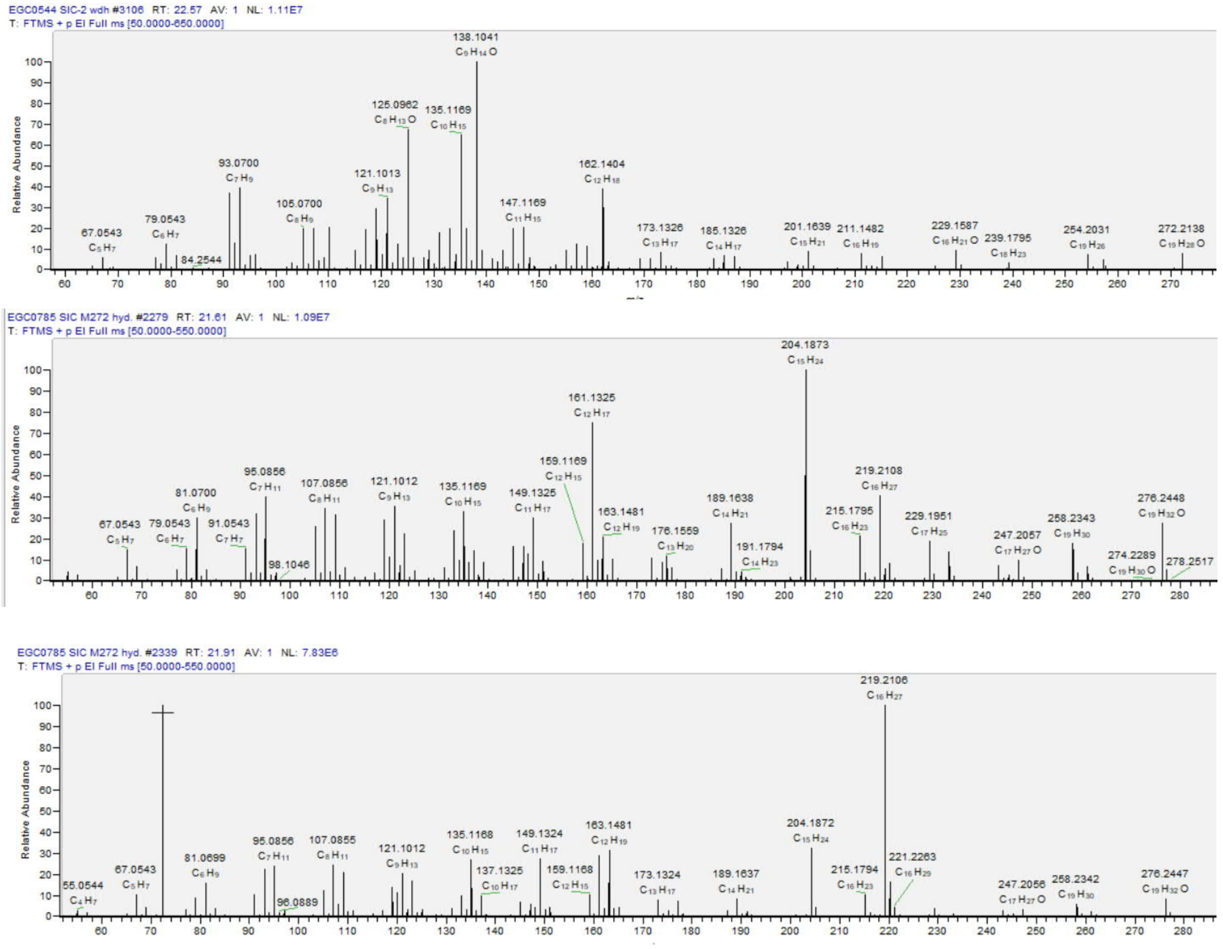

Figure S1. High-resolution mass spectra (GC/Orbitrap) of curvisetone (**1**, top) and hydrogenation products **S1** (middle) and **S2** (bottom).

2. Mass Spectra of Hydrogenated Compound 1

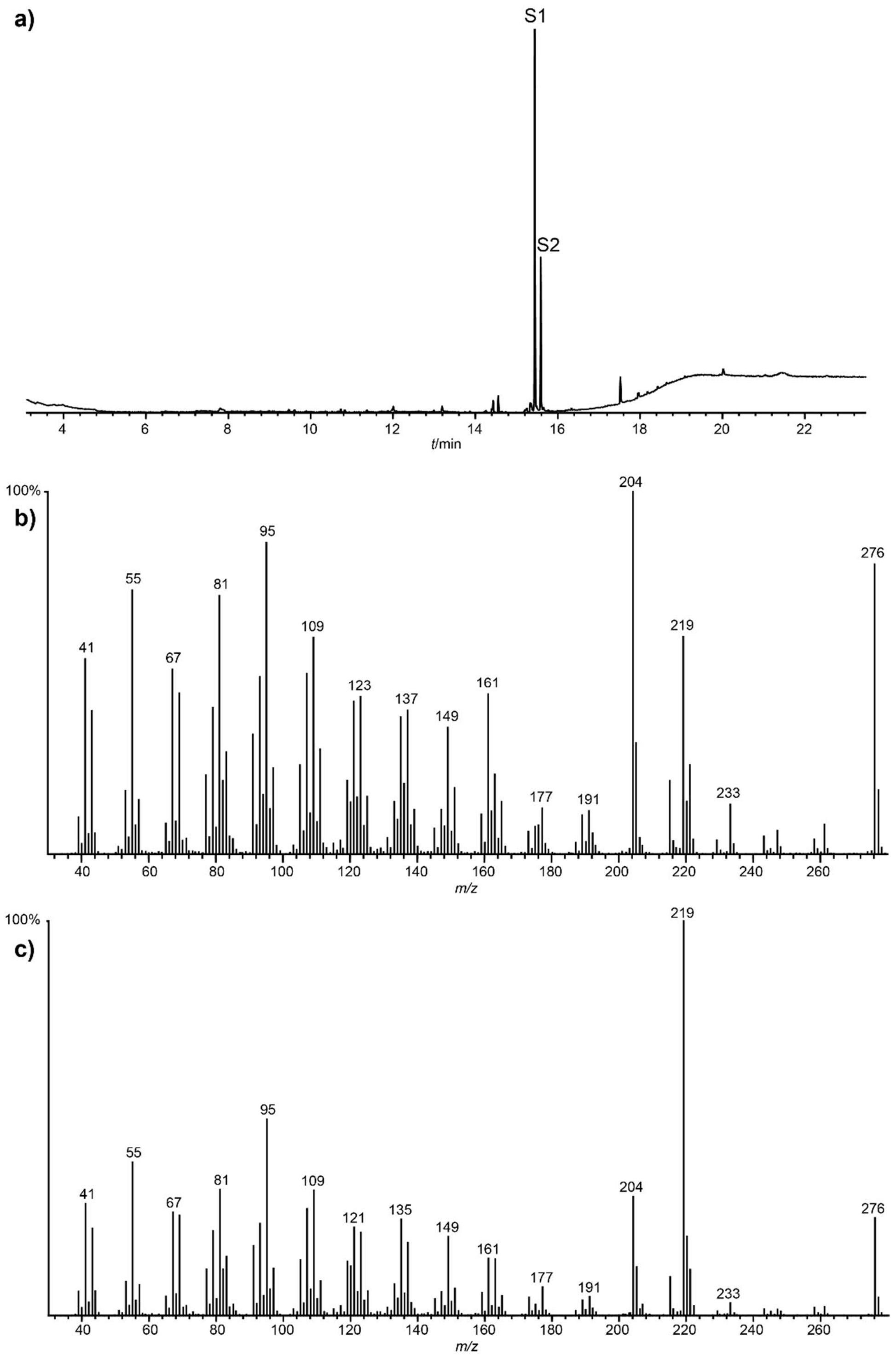

Figure S2. TIC of a hydrogenated pentane extract of *S. curviseta* (a) and mass spectra of hydrogenation products **S1** (b) and **S2** (c).

3. Experimental and Calculated IR Spectra

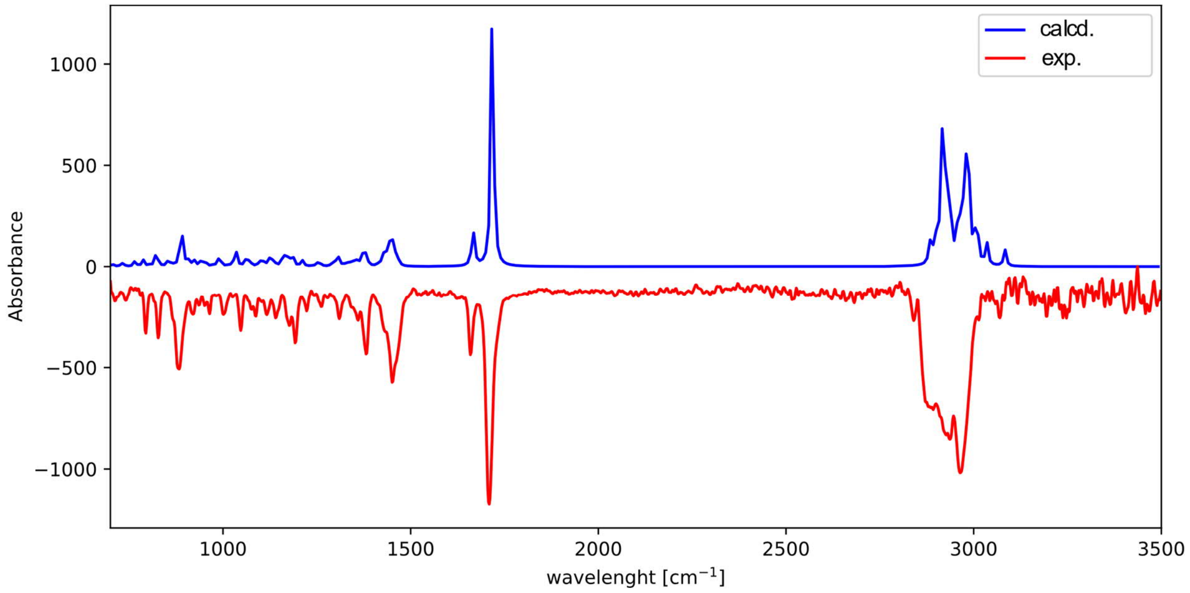

Figure S3. Comparison of the experimental IR spectra of compound **1** (red) and the DFT calculated data (blue) (B3LYP; 6-311+G(d,p)).

4. Time course of Curvisetone Production

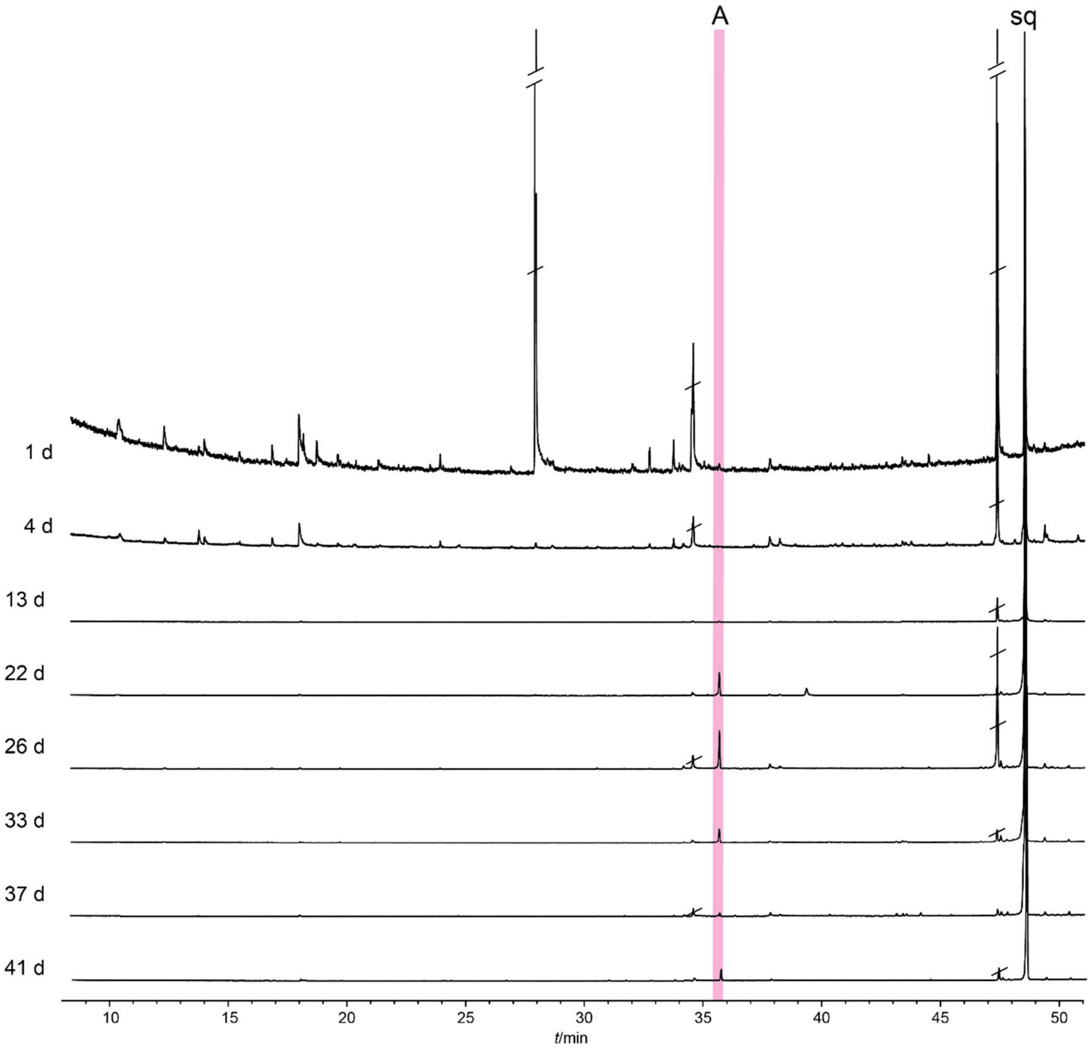

Figure S4. TIC of CH_2Cl_2 extracts of *S. curviseta* at different time points in days after hatching. All chromatograms were scaled to squalene (sq), which was the highest peak in all cases. Impurities are marked by a crossing line.

5. DFT-calculated NMR Data

Table S1. Calculated NMR data for curvisetone (**1**) by DFT (ωB97X-D; 6-31G*) in comparison with the experimental data (see Table 1).

Position	$H_{calc.}$ [ppm]	$H_{exp.}$ [ppm]	$H_{calc.}–H_{exp.}$	$C_{calc.}$ [ppm]	$C_{exp.}$ [ppm]	$C_{calc.}–C_{exp.}$
1a	2.43	2.57	-0.14	37.5	38.47	
1b	2.18	2.40	-0.22			
2				209.6	213.12	-3.5
3	2.27	2.24	0.03	44.7	45.08	-0.4
4	1.92	1.69	0.23	41.3	42.09	-0.8
5a	1.62	1.65	-0.03	32.2	32.04	0.2
5b	1.48	1.44	0.04			
6	2.7	2.82	-0.10	44.0	42.21	1.8
7				145.7	146.29	-0.6
8	5.53	5.38	0.15	120.6	119.52	1.1
9a	2.28	2.39	-0.11	28.7	28.78	-0.1
9b	2.02	2.16	-0.14			
10	1.93	2.02	-0.09	49.1	49.08	0.0
11				38.5	38.67	-0.2
12a	1.96	2.05	-0.09	36.0	36.27	-0.3
12b	1.26	1.41	-0.15			
13				149.4	150.65	-1.3
14a	4.85	4.75	0.10	106.9	104.18	2.7
14b	4.76	4.60	0.16			
15	0.90	0.92	-0.02	13.2	11.50	1.7
16	2.07	2.12	-0.05	32.0	33.18	-1.2
17	1.06	0.99	0.07	23.0	22.50	0.5
18	1.10	1.00	0.10	20.6	21.57	-1.0
19	1.19	1.12	0.07	19.3	18.36	0.9
		MAE:	-0.01		MAE:	-0.07
		RMSE:	0.12		RMSE:	1.34

6. NMR Spectra

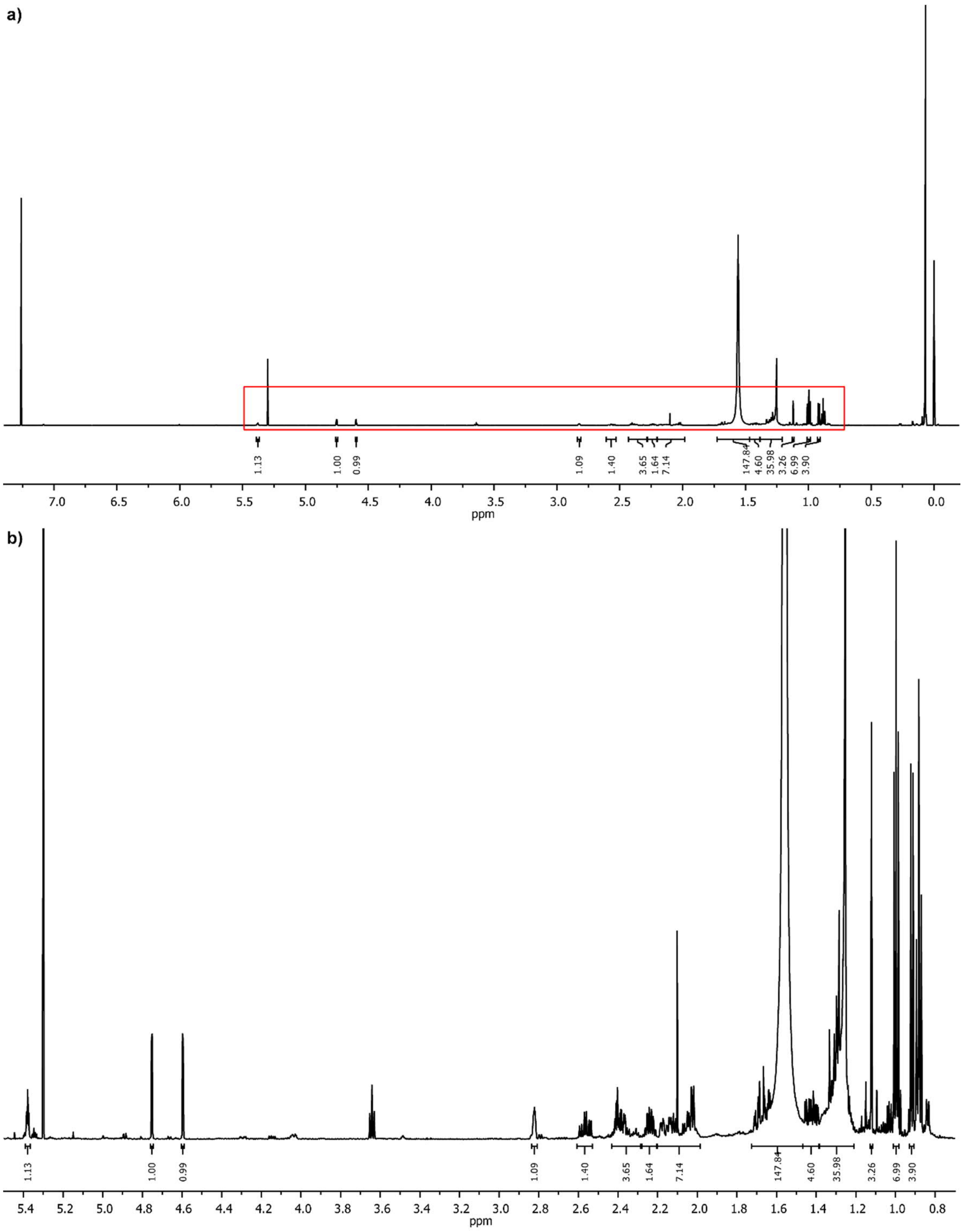

Figure S5. ^{1}H-NMR (600 MHz, $CDCl_3$) of isolated curvisetone (**1**), with the relevant signals (red box) magnified (b).

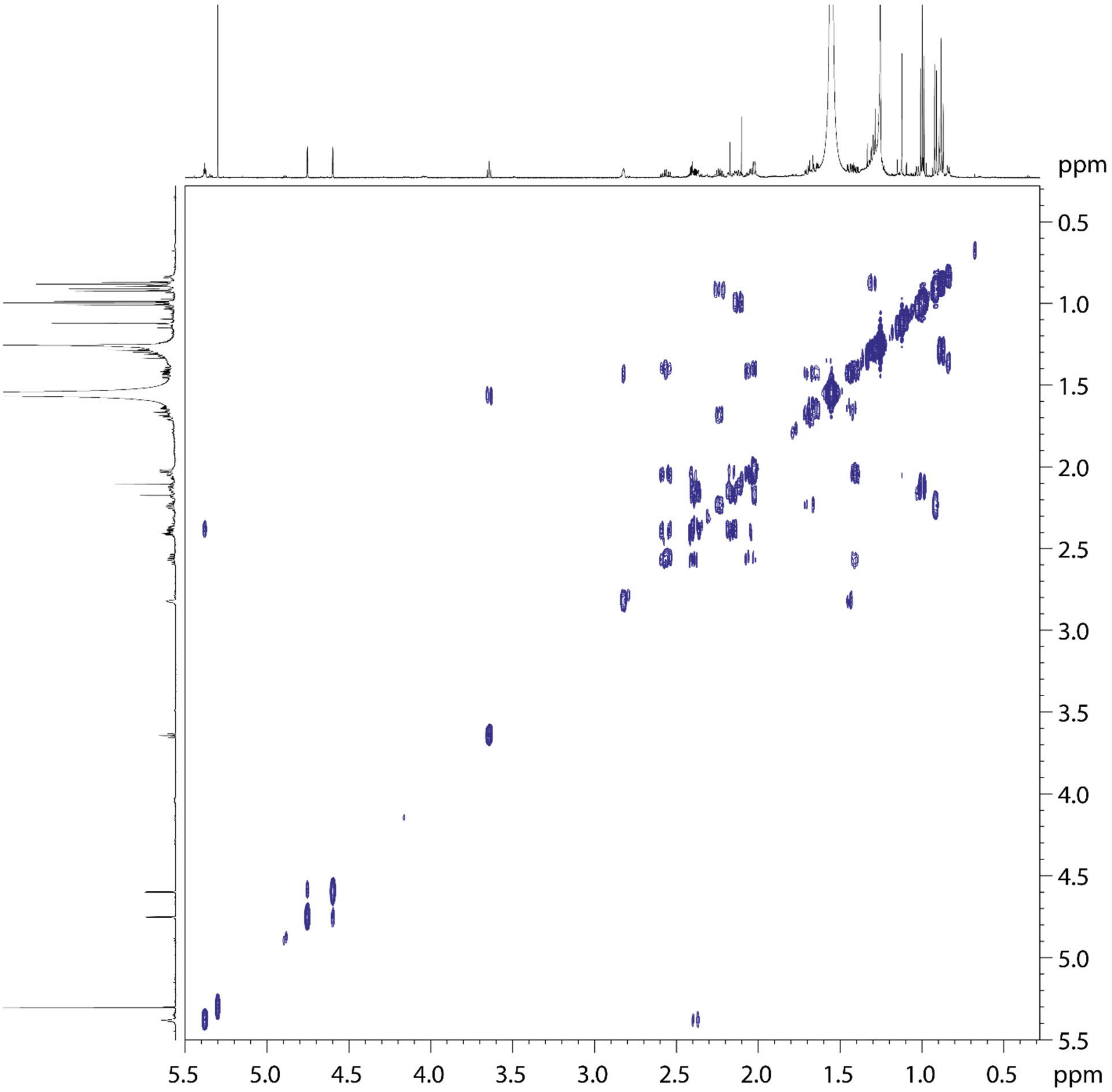

Figure S6. COSY NMR spectrum ($CDCl_3$, ^1H 600 MHz) of compound **1**.

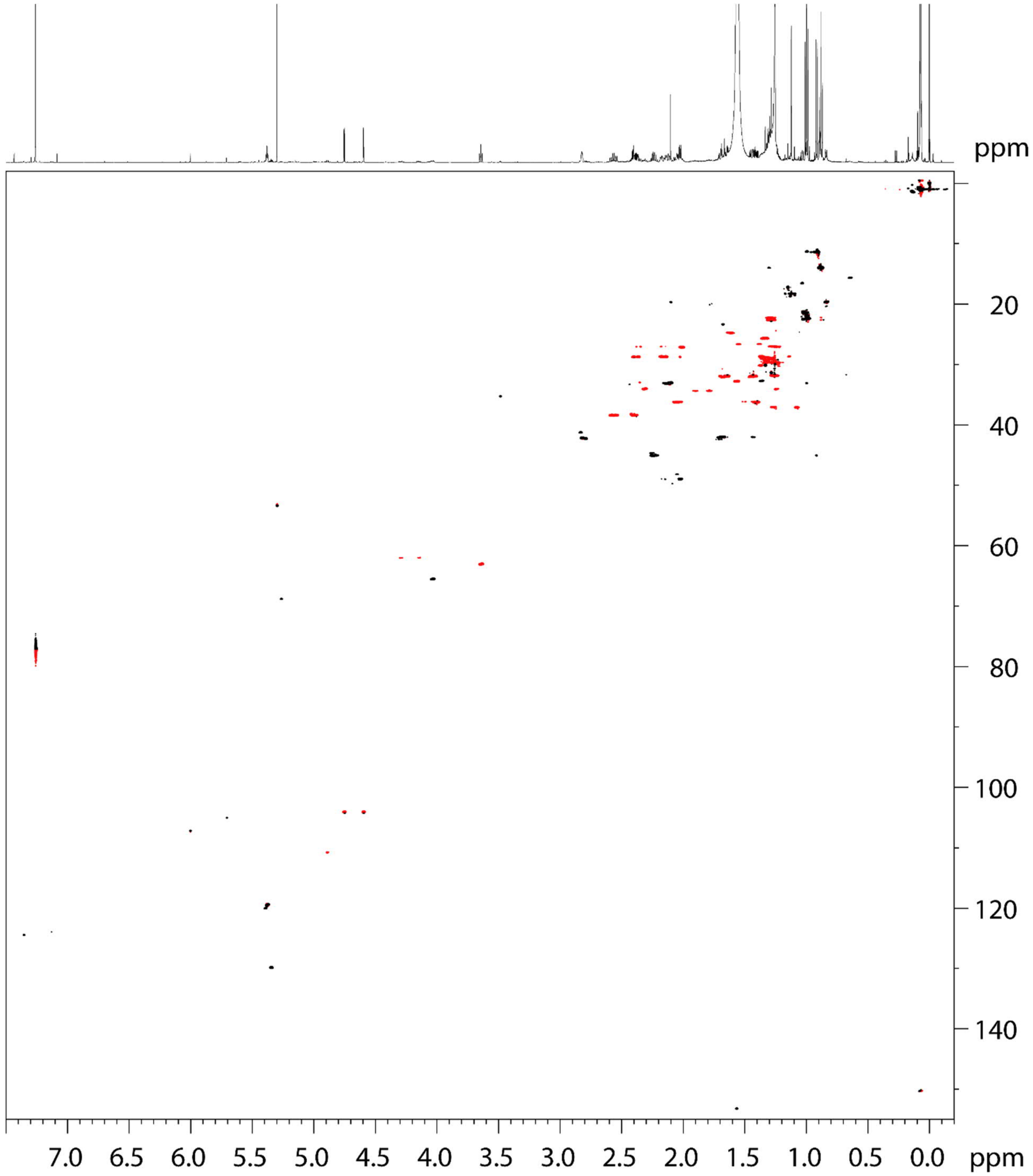

Figure S7. H,C-HSQC NMR spectrum ($CDCl_3$, ^1H 600 MHz, ^{13}C 151 MHz) of compound **1**.

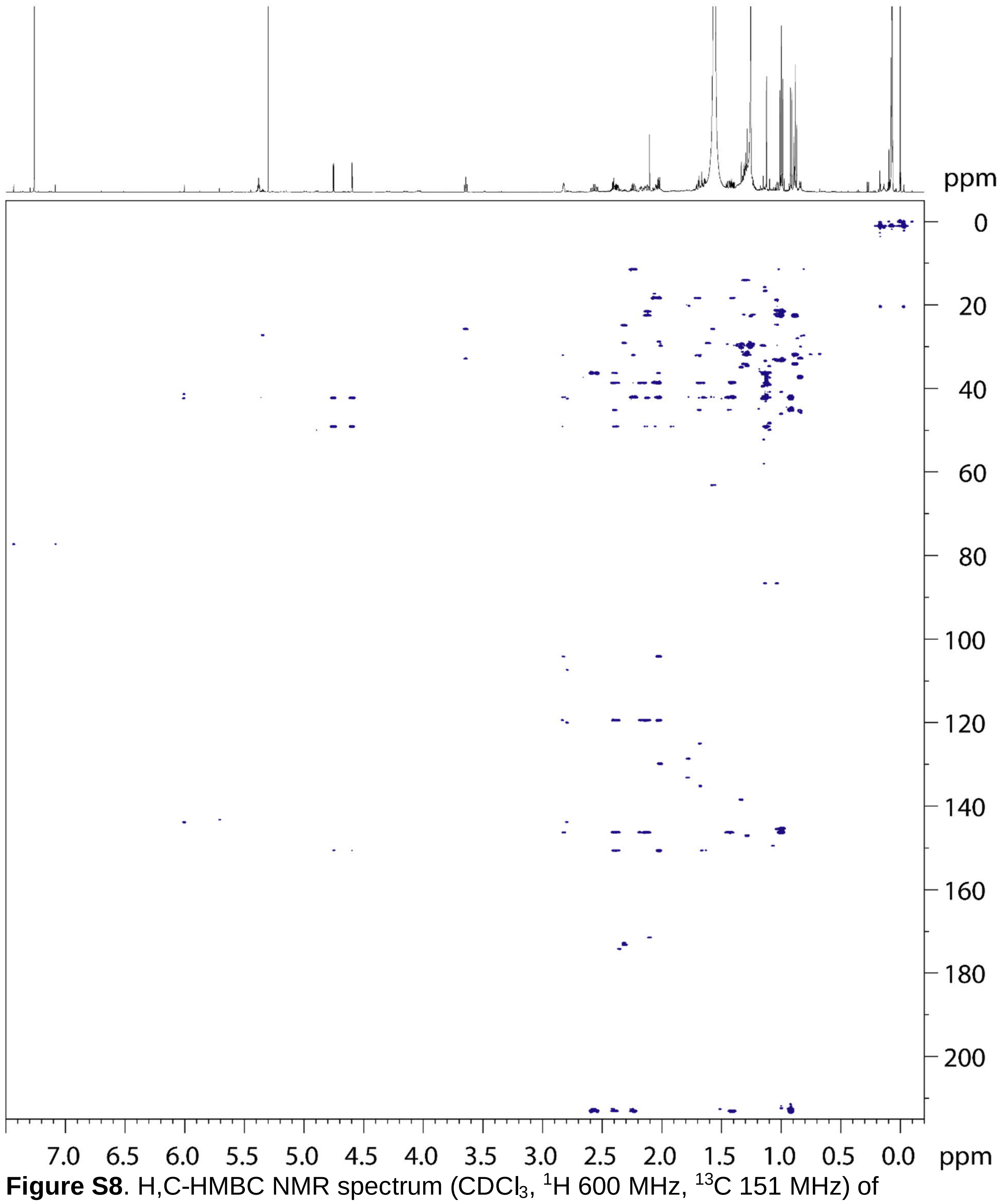

Figure S8. H,C-HMBC NMR spectrum ($CDCl_3$, ^{1}H 600 MHz, ^{13}C 151 MHz) of compound **1**.

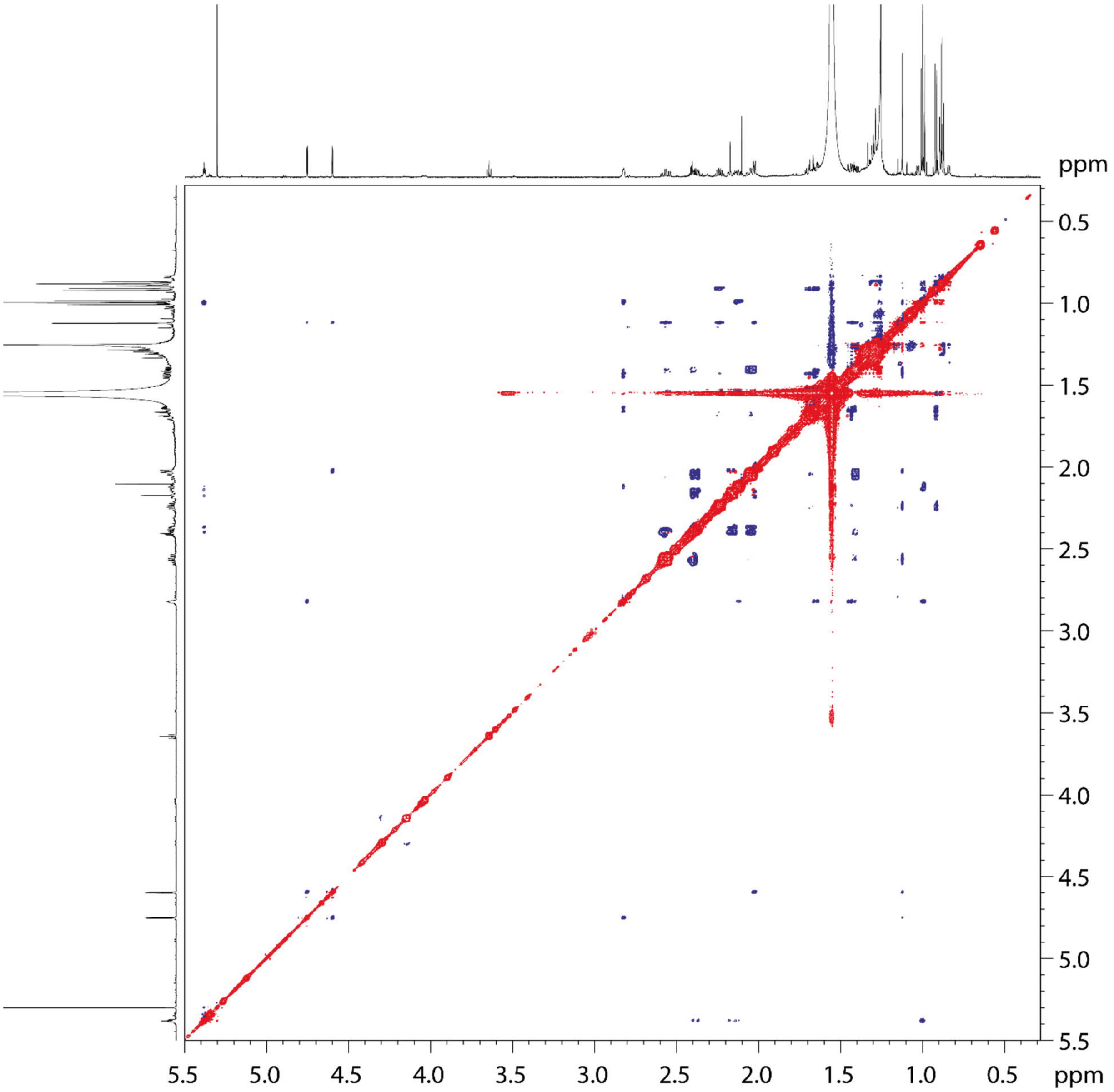

Figure S9. NOESY NMR spectrum ($CDCl_3$, ^1H 600 MHz) of compound **1**.

7. Peak List of the Mass Spectrum of Curvisetone

Name: Scan 4418 (36.236 min): AMN857.D\data.ms
Num Peaks: 203
37 1; 38 2; 39 63; 40 20; 41 220;
42 26; 43 218; 44 14; 45 3; 50 5;
51 25; 52 14; 53 88; 54 19; 55 284;
56 28; 57 30; 58 3; 59 1; 61 1;
62 1; 63 11; 64 6; 65 86; 66 18;
67 169; 68 20; 69 91; 70 7; 71 12;
72 3; 73 2; 74 1; 75 3; 76 4;
77 206; 78 62; 79 224; 80 35; 81 118;
82 26; 83 35; 84 6; 85 7; 86 2;
87 2; 88 1; 89 15; 90 7; 91 742;
92 295; 93 527; 94 54; 95 86; 96 55;
97 37; 98 3; 99 1; 100 1; 101 2;
102 9; 103 63; 104 43; 105 321; 106 51;
107 179; 108 44; 109 135; 110 69; 111 25;
112 1; 113 2; 114 2; 115 127; 116 47;
117 208; 118 53; 119 254; 120 111; 121 338;
122 43; 123 76; 124 28; 125 487; 126 46;
127 28; 128 84; 129 103; 130 37; 131 140;
132 36; 133 218; 134 109; 135 769; 136 440;
137 118; 138 999; 139 148; 140 12; 141 44;
142 32; 143 76; 144 25; 145 149; 146 49;
147 115; 148 40; 149 25; 150 8; 151 9;
152 18; 153 12; 154 7; 155 42; 156 17;
157 81; 158 25; 159 83; 160 24; 161 33;
162 365; 163 60; 164 6; 165 6; 166 2;
167 4; 168 3; 169 21; 170 8; 171 33;
172 11; 173 77; 174 23; 175 23; 176 10;
177 4; 178 1; 179 1; 180 1; 181 3;
182 3; 183 15; 184 6; 185 52; 186 20;
187 62; 188 31; 189 13; 190 2; 191 1;
192 1; 193 1; 195 1; 196 2; 197 11;
198 4; 199 16; 200 16; 201 83; 202 32;
203 6; 204 1; 207 2; 208 1; 209 2;
210 1; 211 40; 212 8; 213 10; 214 5;
215 55; 216 17; 217 4; 218 1; 223 1;
224 1; 225 4; 226 1; 227 2; 228 5;
229 95; 230 19; 231 3; 237 1; 239 26;
240 5; 241 2; 242 1; 243 5; 244 4;
253 2; 254 60; 255 15; 256 3; 257 43;
258 8; 259 1; 270 6; 271 8; 272 128;
273 27; 274 3; 281 1;
