# Supplementary figures and images for: Curvisetone—A Male-Specific Tricyclic nor-Diterpenoid from the Springtail Sinella curviseta
Source: J Nat Prod. 2025 Mar 12;88(3):857–61. doi: 10.1021/acs.jnatprod.4c01432 (PMC11959583; doi:10.1021/acs.jnatprod.4c01432)

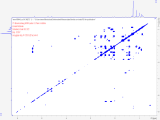

Supplement: Supplementary file 2 — np4c01432_si_002.zip [file np4c01432_si_002.zip › FID for publication/Curvisetone/COSY/pdata/1/thumb.png]
